# Use of multi-trait principal component selection index to identify fall armyworm (*Spodoptera frugiperda*) resistant maize genotypes

**DOI:** 10.3389/fpls.2025.1544010

**Published:** 2025-03-27

**Authors:** Wilber Wambi, Dan Makumbi, Godfrey Asea, Habtamu Zeleke, Anani Y. Bruce, Mulatu Wakgari, Daniel Bomet Kwemoi, Boddupalli M. Prasanna

**Affiliations:** ^1^ African Centre of Excellence for Climate-Smart Agriculture and Biodiversity Conservation, Haramaya University, Dire Dawa, Ethiopia; ^2^ Bulindi Zonal Agricultural Research and Development Institute, National Agricultural Research Organization, Hoima, Uganda; ^3^ Global Maize Program, International Maize and Wheat Improvement Center (CIMMYT), Nairobi, Kenya; ^4^ National Crops Resources Research Institute, National Agricultural Research Organization, Namulonge, Uganda

**Keywords:** Fall armyworm, host plant resistance, multi-trait selection, principal component analysis, selection gain, *Spodoptera frugiperda*, weight-free selection index

## Abstract

The Fall armyworm (FAW), *Spodoptera frugiperda* (J. E. Smith) invaded sub-Saharan Africa (SSA) in 2016 and has since become prevalent in many countries, causing significant maize grain yield losses and reduced grain quality. Breeding for host plant resistance to FAW requires improving multiple traits, complicating selection. This study evaluated the use of principal component (PC)-based multi-trait selection indices to identify FAW resistant maize genotypes. A total of 192 maize hybrids alongside four commercial hybrids, were evaluated over four seasons under artificial FAW infestation. Data on FAW leaf feeding damage (LD) at 7, 14, and 21 days after infestation, and ear damage (ED), ear rot (ER), and grain yield (GY) were recorded. The data were subjected to analysis of variance and PC analysis, and results used to construct two economic weight-free selection indices: PC1-based index (PC1BI) and PC2-based index (PC2BI). Broad-sense heritability estimates were 0.59 to 0.73 for LD, and 0.69 for GY. The two PCs explained 97.1% of the variation among the hybrids. PC1BI, with higher loadings for the leaf feeding damage traits, showed the larger desired gains for these traits (−2.92 to −3.84%) and GY (19.9%), making it a superior index to PC2BI. PC1BI identified six promising hybrids with GY above the cutoff of 7.0 t ha^-1^ for selection under FAW infestation. PC2BI exhibited larger gains for ED (−11.1%) and ER (−45.4%). The index-based selected hybrids consistently outperformed the commercial hybrid checks. The PC-based indices have the potential to serve as valuable tools for breeders to maximize selection gains; however, modifications are necessary to incorporate other desirable agronomic and adaptive traits.

## Introduction

Maize is an important and strategic crop in sub-Saharan African (SSA) for food, feed, and plays key roles in the national economies of several countries as a key industrial and export commodity (Badu-Apraku and Fakorede, 2017; Ekpa et al., 2018, 2019; Erenstein et al., 2022; UBOS, 2022). Sustained production of maize is curtailed by several factors including declining soil fertility, climate change, and emerging pests and diseases (Sisay et al., 2019; Prasanna et al., 2021; Asfaw et al., 2024). The outbreak of the invasive fall armyworm (FAW) pest first reported in 2016 is among the most important pest limiting the production of maize in SSA (Goergen et al., 2016; Otim et al., 2018; Sisay et al., 2019; Tambo et al., 2020). The pest has a broad host range, but its impact is severe on maize fields causing plant damage and yield losses ranging between 20 to 70% especially during aggressive feeding larval stage (Day et al., 2017; Ramirez-Cabral et al., 2017; Montezano et al., 2018). The larval feeding causes several damages to the leaves, stems, and maize ears, further exposing cobs to ear rots and mycotoxin contamination, reducing the quality and safety of grains and their products (FAO, 2017; Pruter et al., 2020; Palumbo et al., 2020; Logrieco et al., 2021). The impact of the pest is mainly severe for smallholder farmers with limited access to inputs, especially pesticides that are costly and require training on use to limit exposure and pesticide risks on humans, crops, livestock and agro-biodiversity (Day et al., 2017; Ramirez-Cabral et al., 2017; Kassie et al., 2020; Tambo et al., 2020; Yan et al., 2022).

The FAW is difficult to control and manage due to its migratory, transboundary and polyphagous behavior, short life cycle and nature of mostly small holder farming in SSA (FAO, 2021; Otim et al., 2021). Host resistance provides a cost-effective, user-friendly, and environmentally safe component of an integrated pest management strategy against FAW under smallholder conditions (Prasanna et al., 2022). Most tropical breeding programs are still in their early stages of host resistance development, and only a few first-generation FAW-tolerant hybrids are commercially available, thus limiting the use of host resistance (Prasanna et al., 2022). Breeding for next-generation and durable resistance demands the consideration of several traits in selection and advancement processes. The key traits targeted by breeders include foliar damage and grain yield traits that benefit the producer, as well as those that influence product quality and consumer acceptance, such as ear damage and ear rot. However, FAW-inflicted leaf-feeding damage and ear quality traits often show an unfavorable correlation with grain yield (Kasoma et al., 2021; Kamweru et al., 2022; Prasanna et al., 2022). Selecting uncorrelated traits focusing on direct selection approaches makes genetic improvement challenging for multiple traits (Olivoto and Nardino, 2020; Zuffo et al., 2020; Batista et al., 2021; Ambrósio et al., 2024). Investigating selection indices aimed at selecting desired second-generation host resistant varieties is crucial given the uncertainty when selection is focused on direct trait approaches. Studies have demonstrated that employing selection indices for multiple traits offers successful chances for breeding several traits into a single genotype (Lopez-Cruz and de los Campos, 2021; Padjung et al., 2021; Anshori et al., 2022; Cerón-Rojas and Crossa, 2022; Farid et al., 2022; Anshori et al., 2024). Breeders utilize a variety of selection indices, such as the Smith-Hazel index (Smith, 1936), base index (Williams, 1962), restricted selection index (Kempthorne and Nordskog, 1959), and the Tai index (Tai, 1977). Some of these indices require the use of economic weights but economic weights for traits are subjective and not easy to determine. Selection indices that require weights generated intrinsically from data are easy to use and amenable for breeders. An example of such an index is the principal component (PC) based index which is possibly the most used (Agudelo-Gómez et al., 2016; Tang et al., 2021; Padjung et al., 2021). Principal component analysis is useful in eliminating redundancy in univariate analyses (Iezzoni and Pritts, 1991), and therefore, PC-based indices also eliminate multicollinearity among variables. The PC-based index method has been used to identify maize varieties with acceptable nutritional value and promising physiological seed quality traits (Tang et al., 2021; Padjung et al., 2021). Principal component indices could thus be useful when selecting FAW-resistant genotypes since some of the traits are scored multiple times, and others show unfavorable correlations. The objective of this study was to evaluate PC-based indices for the selection of maize hybrids with resistance to FAW and improved grain yield under artificial infestation conditions.

## Materials and methods

### Plant materials

In 2019, nineteen (19) inbred lines were crossed in a diallel mating scheme to generate 171 F1 test hybrids without reciprocals. The inbred lines were developed by CIMMYT and showed promising host responses to FAW feeding damage under artificial infestation in preliminary tests that were done in 2017 and 2018. **Table 1** details characteristics, pedigree, and origin of parental lines utilized in the half-diallel crosses. The crosses were made at Kenya Agricultural and Livestock Research Organization (KALRO) Kiboko Research Station (2°15’S, 37°75’E, 975 m a.s.l) in Kenya. In addition to the diallel hybrids, 17 experimental hybrids, including two internal genetic gain checks (CKH10769 and CKH10717), and two commercial check hybrids (WH401 and WH505) were included in the study.

**Table 1:** List and characteristics of parental lines utilized in the half diallel crossing.

Code	Name	Pedigree	Characteristics	Origin
1	CML247	(G24-F119/G24-F54)-6-4-1-1-B-B-B-B	Tolerant to ear rot	Mexico
2	CML269	P25STE-C1-FS13-6-1-1-#-B-B-B-B	Tolerant to ear rot	Mexico
3	CML17	P22TSR-B-B-40-2-1-2-B-B-B-B	Lodging resistant	Mexico
4	CML23	P25-FS112-2-2-2-1-4-B-B-B-B	Tolerant to ear rot	Mexico
5	CML274	(AC7643/P43-F7)-2-3-4-3-B-B-B-B	Tolerant to ear rot	Mexico
6	CML371	MBRETW-F2-56-1-1-1-B-B-6-B-B-B-B	Multiple borer resistance	Mexico
7	CML372	MBRETW-F2-177-3-1-1-B-B-B-B	Multiple borer resistance	Mexico
8	CML402	AC8222-6-2-2-B-#-#-2-B*3-1-B-B-B-B	Tolerant to ear rot	Mexico
9	CML476	P21MRRS-C1-525-1-B-B-B-B	Susceptible to FAW	Mexico
10	DL187008	MDRC3 Bc/MBRC5 Bc F59-1-B-#-1-2-B-B-B-B	Multiple borer resistance	Kenya
11	DL187009	MBR/MDRC4 Bc F34-1-B-#-1-1-B-B-B-B	Multiple borer resistance	Kenya
12	DL187010	MIRTC5 Bco F62-2-2-1-1-2-1-B-B-B-B	Multiple insect resistance	Kenya
13	DL187011	P84c3BcxMIRTC5 Bco F10-1-2-2-2-3-1-B-B-B-B	Multiple insect resistance	Kenya
14	DL187012	P84c3BcxMIRTC5 Bco F80-4-2-1-4-1-1-B-B-B-B	Multiple insect resistance	Kenya
15	DL187019	MBRC6 Bc F234-1-B-#-1-1-B-B-B-B	Multiple borer resistance	Kenya
16	CML334	P590-C3-F374-2-1-2-B-#-3-3-B-B-B-B	Southwestern corn borer resistance	Mexico
17	CKSBL10153	[(MIRTC4Am F128-B-1-3-B-B x CL-02450)-B]-1-B-B-B-B	Stem borer tolerance, FAW tolerance	Kenya
18	CML71	ANTGP2-5-#-1-2-1-1-5-5-7-B-B-B-B	Tolerant to FAW	Mexico
19	CML345	P390SCB-C1-F72-1-1-1-1-#-6-B-B-B-B	Tolerant to FAW	Mexico

### Evaluation of response to FAW infestation

A total of 171 diallel hybrids were evaluated along with 15 or 17 experimental hybrids, two internal genetic gain checks (CKH10769 and CKH10717), and two commercial check hybrids (WH401 and WH505) for four seasons under artificial FAW infestation. In 2020, the trial included 192 hybrids, while in 2021, there were 190 hybrids. The experimental design was a 4 × 48 alpha-lattice in 2020 and a 5 × 38 alpha-lattice in 2021, each with two replications. The experiments were conducted in screen houses in Kiboko, Kenya. The hybrids were planted in a single-row plot, 3 meters long, with 0.75 meters between rows and 0.25 m between plants. FAW neonates, obtained from the CIMMYT insectary at KALRO Katumani Agricultural Experimental Station in Machakos County, Kenya, were used to artificially infest the hybrids. The rearing of FAW neonates followed the protocols described in detail by Prasanna et al. (2018) and Anyanda et al. (2022). Seven neonates were placed in the whorl of each plant at the V5 stage using a camel brush, following the protocols outlined by Prasanna et al. (2018).

### Data collection

Fall armyworm leaf feed damage (LD) was assessed at 7 (LD1), 14 (LD2), and 21 (LD3) days after artificial infestation using a visual rating scale of 1–9 (Davis and Williams, 1992; Prasanna et al., 2018). A score of 1 is considered highly resistant, 2 to 3 resistant, 4 to 5 moderately resistant, 6 to 7 susceptible, and 8 to 9 highly susceptible. The average leaf feeding damage score (LD_AV) was computed from the three ratings. At harvest, ear damage (ED) was also rated on a scale of 1–9 (Prasanna et al., 2018; Kamweru et al., 2022), where 1 indicated no ear feeding damage and 9 represented severe ear feeding damage. Ear rot (ER) was recorded by counting rotten ears per plot after harvest, expressed as a percentage of the total harvested ears. All the harvested ears were then weighed to determine plot field weight data. Grain moisture content was measured using a moisture meter. Using field weight, grain moisture content, and an average shelling percentage of 80%, grain yield (GY, t ha^-1^) was calculated and adjusted to 12.5% moisture content.

### Data analyses

#### Analysis of variance

Data were checked for normality using the Shapiro-Wilk test by examining residual plots, and histograms. The data were found to be normally distributed, and therefore no data transformation was required. A linear mixed model was used to perform analyses of variance (ANOVA) across environments using the META-R software (Alvarado et al., 2020). The linear mixed model used for the analysis of the data was:


$${\text{Y}}_{ijkl}=µ+{\text{G}}_{i}+{\text{E}}_{j}+{\text{R}}_{k}\left({\text{E}}_{j}\right)+{\text{B}}_{l}{\left(\text{ER}\right)}_{jk}+{\text{GE}}_{ij}+\epsilon_{ijkl}$$

where Y*_ijk_* is the response variable; µ is the intercept; G*_i_* is the effect of the *i*th genotype; E*_j_* is the effect of the *j*th environment; R*_k_* (E_j_) is the effect of *k*th replicate in the *j*th environment; B*_l_* (ER)*_jk_* is the effect of the *l*th block within the *k*th replicate at the *i*th environment; GE*_ij_* is the effect of the interaction between the *i*th genotype and the *j*th environment; and ϵ_ijkl_ is the experimental error associated with the i*th* genotype, j*th* environment, k*th* replicate and l*th* block. The error is assumed to have a mean zero and a homoscedastic variance $\sigma_{\epsilon }^{2}$ . The best linear unbiased estimates (BLUEs) computed from a combined ANOVA were used to create the indices and perform correlation analysis. Broad-sense heritability was estimated for combined environments using variance components following Hallauer et al. (2010) as:


$$H^{2}=\frac{\sigma_{G}^{2}}{\sigma_{G}^{2}+\frac{\sigma_{GE}^{2}}{e}+\frac{\sigma_{E}^{2}}{er}}$$

where $\sigma_{G}^{2}$ is the genotypic variance, $\sigma_{GE}^{2}$ is the genotype × location interaction variance, e is the number of environments, r is the number of replicates, and the $\sigma_{E}^{2}$ is the residual variance.

### Construction of principal component indices for hybrid selection

The data for six FAW-resistance-related parameters (LD1, LD2, LD3, ED, ER, and GY) from the 192 (2020) or 190 (2021) maize genotypes were standardized to have a mean of zero and variance of one for each trait. The standardized values were then subjected to Principal Component (PC) analysis to obtain weights for constructing the selection indices using the META-R software. The decision on the number of PCs to use in the index was informed by analysis of the scree plot, where only PCs with eigenvalues greater than one were selected (**Supplementary Figure 1**). This criterion led to selection of the first two principal components (PC1 and PC2) used in the study. The index score values for each genotype were calculated by summation of the products of the BLUEs across environments for each trait and their respective weights (eigen vectors) on the first two principal component axes (PC1 and PC2) following the procedures outlined by Tang et al. (2021). The computations were done in Microsoft Excel, considering a set of all six FAW resistance-related traits. Two PC-based selection index schemes were developed: a PC1-based index (PC1BI) and a PC2-based index (PC2BI).

The model for constructing PC1BI was:


PC1BI=−0.95×LD1+−0.93×LD1+−0.91×LD3+−0.50×ED+−0.70×ER+1.00×GY

while the model used to construct PC2BI was:


PC2BI=−0.36×LD1+−0.44×LD2+−0.45×LD3+1.00×ED+0.83×ER+−0.08×GY

The LD1, LD2, LD3, ED, ER, and GY correspond to the hybrid means for leaf feeding damage score at 7, 14, and 21 days after infestation, ear damage score, ear rot score, and grain yield, respectively. The multiplicative factors for each trait were the eigen vectors from the PC analysis. The genotypic correlations among traits and the index score values were computed and visualized using the R package ‘*psych*’ version 2.4.6 (William, 2024).

### Performance of PC-indices and hybrid selection

To evaluate the selection efficiency of the PC-based indices, multi-trait selection gains among seven FAW resistance-related traits either through conventional single-trait or principal component-based index selection options were compared. Each trait, including LD1, LD2, LD3, LD_AV, ED, ER, and GY, was considered as an independent conventional single-trait direct selection option. In each selection scheme, the hybrids were ranked and the average of the 15 or 10 top-performing selected hybrids was considered while estimating the selection deferential for the traits. The selection gain (GS) for each trait was calculated as a percentage of the selection differential, expressed relative to the grand mean of the 192 hybrids, and then multiplied by the trait heritability (Santos et al., 2018; Zuffo et al., 2020; Norman et al., 2022). Accordingly, the equation used to estimate selection gain (GS) was:


$$GS=\frac{\left(Xs-Xo\right)\times H^{2}}{Xo}$$

where, Xs  is mean of the 15 or 10 top-performing selected hybrids, Xo is the grand mean of the 192 hybrids, (Xs−Xo)=selection deferential,  and $H^{2}$ is the broad-sense heritability of the trait.

Additionally, the relative gain, estimated as RG = [(Genotype–Check)/Check] × 100 was used to compare the performance of the 15-best index-selected hybrids against the commercial checks WH401 and WH505 following the method of Matias et al. (2020). A grain yield of 7 t ha^-1^ was used as the cutoff, based on the trait metrics developed and used in the Stage gate advancement process by the Global Maize Program of CIMMYT for Product Profile #1 (intermediate maturity maize). Relationship heatmaps based on the top 15 and five bottom index-selected hybrids were generated using the ‘pheatmap’ package in R to visualize and identify superior genotypes that exhibited desired combinations for several traits (Kolde, 2019).

## Results

### Analysis of variance, variance components, heritability and genotype mean performance

A combined ANOVA revealed significant (*P*< 0.01) environment (E), genotype (G), and G × E mean squares for all traits (**Table 2**). The genotypic variance ( $\sigma_{G}^{2}$ ) and G × E variance ( $\sigma_{GE}^{2}$ ) were of similar magnitude for LD1 but $\sigma_{G}^{2}$ was larger than $\sigma_{GE}^{2}\;$ for the other traits (**Table 3**). The broad-sense heritability (*H* ^2^) estimates were moderate for LD1, LD3, ED, ER, and GY (range 0.59 to 0.69) and high (range 0.73 and 0.79) for LD2 and LD_AV. The mean LD across the three scores was 5.1 while the mean GY was 5.1 t ha^-1^ for the trial (**Table 3**). The performance of the test hybrids and checks is presented in **Supplementary Table 1**. A total of 186 hybrids had leaf feeding damage 1 (LD1) scores of<4, while six hybrids showed LD1 scores >4. For LD2, moderate resistance scores ranged from 5.4 (E163) to 5.9 (E82) for 66 hybrids, while for LD3 the scores ranged from 5 (E152) to 5.9 (E94) for 148 hybrids. Fifty-four experimental hybrids recorded average leaf feeding damage (LD_AV)<5, with check hybrids WH401 and WH505 showing LD_AV >6. For ear damage, all the genotypes recorded ear damage scores<3. Of the test genotypes, 141 experimental hybrids had ER<10%, while the check entries exhibited ER between E192 (17.4%) to E191 (31.3%). Grain yield for the experimental hybrids under artificial FAW infestation ranged from 2.2 to 7.8 t ha^-1^, with 11 hybrids yielding ≥ 7 t ha^-1^. The best check WH505 yielded 4.4 t ha^-1^.

**Table 2:** Combined analysis of variance for FAW resistance parameters and agronomic traits of 192 hybrids evaluated under artificial FAW infestation in 2020-2022.

Source of variation	df	LD1	LD2	LD3	LD_AV	ED	ER	GY
Environment (E)	3	42.82***	38.08***	168.89***	20.16***	88.33***	4023.00***	982.60***
Rep (E)	4	9.47***	18.97***	18.33***	6.70***	4.09***	420.00***	60.90***
Block (Rep/E)	336	0.24***	0.45***	0.68***	0.25***	0.30***	33.00**	4.50***
Genotypes	191	0.46***	0.59***	0.88***	0.42***	0.62***	86.00***	11.60***
Genotypes × E	565	0.22***	0.28***	0.47***	0.16***	0.30***	40.00***	4.30***
Residual	422	0.12	0.11	0.27	0.70	0.17	25.00	1.70

**, *** Significant at *P* < 0.01 and *P* < 0.001, respectively.

LD1, LD2, and LD3, leaf feeding damage score at 7, 14, and 21 days after infestation; LD_AV, Average leaf feeding damage score; ED, Ear damage; ER, Ear rot; GY, Grain yield.

**Table 3:** Variance component and broad-sense heritability (*H* ^2^) estimates and means for FAW resistance parameters and agronomic traits of 192 hybrids evaluated under artificial FAW infestation in 2020-2022.

Traits	$\sigma_{G}^{2}$	$\sigma_{GE}^{2}$	$\sigma_{e}^{2}$	*H* ^2^	Mean	Range	LSD_0.05_	CV (%)
LD1	0.03***	0.02***	0.12	0.62	3.52	2.90 – 4.80	0.33	9.71
LD2	0.04 ***	0.01ns	0.11	0.73	6.04	5.40 - 7.30	0.33	5.53
LD3	0.05***	0.00ns	0.27	0.59	5.72	5.00 – 6.90	0.42	9.12
LD_AV	0.04***	0.00ns	0.07	0.79	5.09	4.60 – 6.20	0.27	5.28
ED	0.04***	0.02*	0.18	0.59	1.98	1.40 – 2.90	0.37	21.27
ER	11.03***	3.33	28.80	0.65	8.14	1.10 – 31.30	5.71	65.91
GY	0.88***	0.72***	1.70	0.69	5.11	2.20 – 7.80	1.55	25.55

*, *** Significant at *P* < 0.05 and *P* < 0.001, respectively. $\sigma_{G}^{2}$ , genotypic variance; $\sigma_{GE}^{2}$ , variance of genotype x environment interactions; $\sigma_{e}^{2}$ , error variance; LD1, LD2, and LD3, Leaf feeding damage score at 7, 14, and 21, days after infestation; LD_AV, Average leaf feeding damage score; ED, Ear damage; ER, Ear rot; GY, Grain yield.

### Principal component analysis, correlations among FAW resistance parameters, and index scores

**Figure 1:**
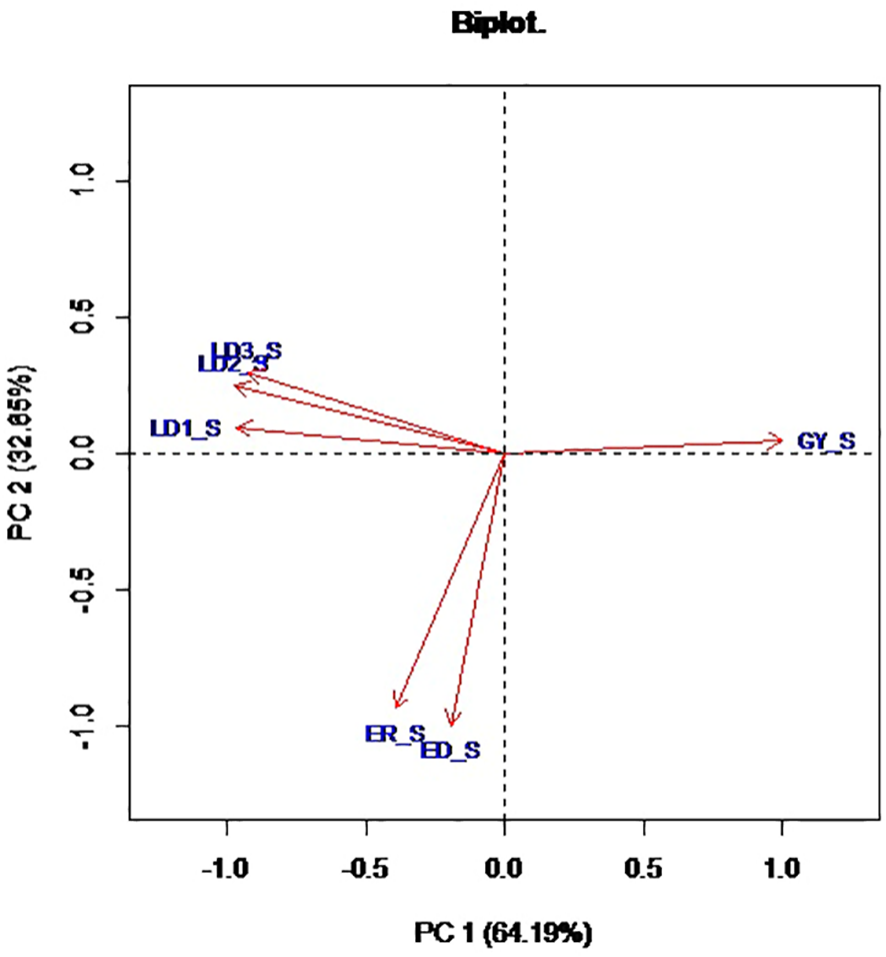
Biplot of the first two principal components for FAW resistance parameters and agronomic traits of 192 hybrids. LD1, LD2, LD3 = Leaf feeding damage score at 7, 14, 21 days after infestation, respectively; ED, Ear damage; ER, Ear rot; GY, Grain yield.

**Figure 2:**
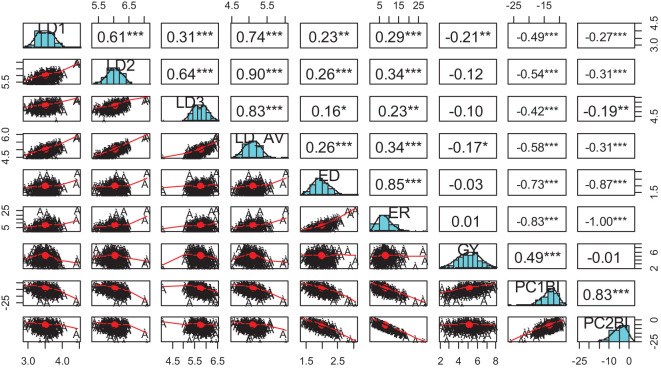
Genotypic correlations between FAW resistance parameters and index score values. LD1, LD2, LD3 = Leaf feeding damage score at 7, 14, 21 days after infestation, respectively; ED, Ear damage; ER, Ear rot; GY, Grain yield; PC1BI, Principal component 1-based index; PC2BI, Principal component 2-based index. *, **, *** Significant at *P* < 0.05, *P* < 0.01, and *P* < 0.001, respectively.

Principal component analysis revealed that the first two PCs explained 97.1% of the total variation among the hybrids (**Table 4**; **Figure 1**). The traits with the highest loadings on PC1 (64.2%) were the leaf-feeding damage scores and grain yield. The traits with the highest loadings on PC2 (32.9%) were ear damage and ear rot. The genotypic correlations between the time series FAW leaf feeding damage traits LD1, LD2, LD3, and LD_AV were strong (*r* = 0.3 to 0.9) (**Figure 2**). The PC1BI and PC2BI index values for the hybrids are presented in **Supplementary Table 1**. The desirable index value is the larger value. The PC1BI index values ranged from -26.3 to -8.7, for WH505 and E154, respectively. For PC2BI, the index values ranged from -27.6 to 1.4 for hybrids WH505 and E28, respectively. The PC1BI and PC2BI index values were strongly correlated with all traits except GY with PC2BI values.

**Table 4:** Eigenvectors of the first two principal component axes (PC1 and PC2) based on correlation matrix of FAW resistance parameters.

Traits	PC1	PC2
Leaf feeding damage score at 7 days (LD1)	**-0.97**	0.10
Leaf feeding damage score at 14 days (LD2)	**-0.98**	0.25
Leaf feeding damage score at 21 days (LD3)	**-0.93**	0.30
Ear damage	-0.19	**-1.00**
Ear rot	-0.39	**-0.94**
Grain yield	**1.00**	0.05
Proportion of variance explained	64.2%	32.9%

Bold values indicate loadings above 50% on each PC.

### Performance of PC-based indices and hybrid selection

**Figure 3:**
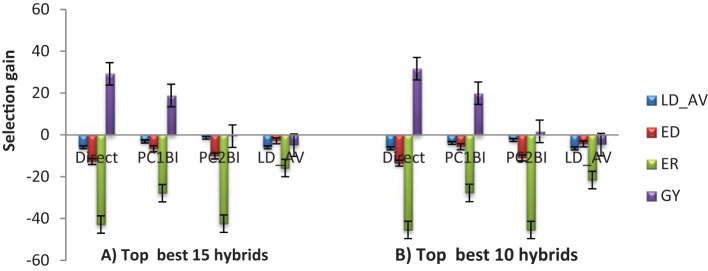
Selection gain under PC and direct-based selection schemes based on: **(A)** top 15 **(B)** top 10 selected hybrids. LD_AV, Average leaf feeding damage score; ED, Ear damage; ER, Ear rot; GY, Grain yield.

**Figure 4:**
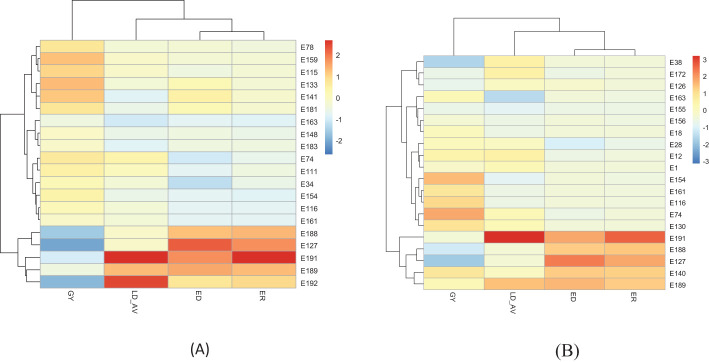
Heatmaps displaying relationships between FAW resistance parameters and genotypes based on top 15 and bottom 5 hybrids selected using: **(A)** PC1BI and **(B)** PC2BI-selected hybrids. On the scale, deep red color was desirable for traits that were desired for increase, while deep blue was required for traits that were desired for decrease.

The expected selection gains for FAW resistance parameters and agronomic traits based on the PC indices are presented in **Table 5** and **Figure 3**. Using the PC1BI resulted in the desired genetic gains for all traits for the top 10 and 15 selected hybrids. A similar result was obtained using PC2BI except for GY when the top 15 hybrids were selected. There were similar gains in the desired direction for the traits with high loadings on PC1 with selection using PC1BI. The selection gain for GY was higher (19.9%) under PC1BI compared with PC2BI (1.68%) when selecting the top 10 hybrids. Index PC2BI, based on high PC2 loadings on ED and ER, showed larger gains for these traits relative to PC1BI. Direct selection based on average leaf feeding damage (LD_AV) showed larger gains for LD1, LD2, LD3, and ER compared to PC1BI, direct selection based on ER (ER_DS), and direct selection based on GY (GY_DS). Direct selection based on GY resulted in larger gains for GY but undesirable direction for ED and ER. The ranking of the top 15 and bottom five experimental hybrids under different selection schemes is shown in **Table 6**. The LD of the top 10 hybrids ranged from 4.6 to 5.2, while their grain yield ranged from 5.1 to 7.7 t ha^-1^. Six of the top 15 based on PC1BI yielded > 7 t ha^-1^. Out of the top 15 hybrids based on PC1BI, five hybrids (E116, E154, E161, E163, and E74) were ranked among the top 15 hybrids selected based on PC2BI while four hybrids (E141, E148, E163, and E183) were among the top under direct selection based on LD_AV. Hybrid E163 was consistently among the top 15 through all three selection schemes. Two genotypes E155 and E163 were selected under both PC2BI and LD_AV selection schemes. The percentage relative selection gain of each of the 15 top hybrids against the commercial checks WH401 and WH505 under different selection schemes showed that the hybrids would result in larger relative gains compared to the two commercial checks (**Table 7**). The gains were mainly observed for LD_AV, ED, ER, and GY, with the larger magnitude gains recorded for the PC-based selection indices. The relationship heatmaps (**Figure 4**) categorized the genotypes into two main groups. One cluster included hybrids that clustered together with the check varieties characterized with poor trait-genotype associations, while the other had 15 index-based selected genotypes that were divided into subgroups for each index scheme. Under PC1BI, one subgroup comprised six top yielding hybrids (E78, E159, E115, E133, E141, and E181) that exhibited strong association with GY (**Figure 4A**). Seven hybrids (E74, E111, E34, E154, E116, E116, and E163) in second subgroup combined relatively higher GY and lower ER and ED damage under the same index. Out of the seven, four hybrids (E116, E154, E161, and E163) combined higher GY and lower LD_AV scores, and one E174 combined higher GY and lower ED scores under PC2BI (**Figure 4B**).

**Table 5:** Principal component-based index (PC1BI and PC2BI) and direct selection gains and direction of selection scenarios for FAW resistance parameters and agronomic traits.

Selection scheme	Traits	Selection gains	Direction	Selection gains	Direction
		Top 10 selected hybrids		Top15 selected hybrids	
PC1BI					
	LD1	-3.43	+	-2.95	+
	LD2	-2.92	+	-2.14	+
	LD3	-3.04	+	-2.66	+
	LD_AV	-3.84	+	-3.13	+
	ED	-5.68	+	-6.62	+
	ER	-27.77	+	-27.89	+
	GY	19.90	+	18.83	+
PC2BI					
	LD1	-2.81	+	-1.69	+
	LD2	-1.84	+	-0.96	+
	LD3	-1.66	+	-0.94	+
	LD_AV	-2.43	+	-1.33	+
	ED	-11.05	+	-10.08	+
	ER	-45.43	+	-42.43	+
	GY	1.68	+	-0.63	–
LD_AV_DS					
	LD1	-5.33	+	-5.51	+
	LD2	-5.08	+	-4.63	+
	LD3	-5.21	+	-4.49	+
	LD_AV	-6.46	+	-5.92	+
	ED	-4.31	+	-2.86	+
	ER	-21.58	+	-15.86	+
	GY	-4.64	–	-4.92	–
ED_DS					
	LD1	-0.45	+	-1.15	+
	LD2	-0.09	+	-0.76	+
	LD3	-0.99	+	-1.06	+
	LD_AV	-0.71	+	-1.21	+
	ED	-13.53	+	-12.77	+
	ER	-36.84	+	-33.84	+
	GY	2.60	+	1.62	+
ER_DS					
	LD1	-2.81	+	-1.85	+
	LD2	-1.84	+	-1.47	+
	LD3	-1.66	+	-2.08	+
	LD_AV	-2.43	+	-2.19	+
	ED	-11.05	+	-9.05	+
	ER	-45.43	+	-42.85	+
	GY	1.68	+	-2.50	–
GY_DS					
	LD1	-1.51	+	-1.11	+
	LD2	-1.18	+	-0.72	+
	LD3	-1.82	+	-1.84	+
	LD_AV	-1.85	+	-1.54	+
	ED	1.78	–	1.33	–
	ER	0.78	–	3.67	–
	GY	31.65	+	29.17	+

+, Desired; -, Undesirable; PC1BI, Principal component 1-based index; PC2BI, Principal component 2-based index; and LD_AV_DS, ED_DS, ER_DS, and GY_DS = direct selection schemes based on average leaf feeding damage, ear damage, ear rot and grain yield, respectively.

LD1, LD2, LD3, Leaf feeding damage score at 7, 14, 21 days after infestation, respectively; LD_AV, Average leaf feeding damage; ED, Ear damage; ER, Ear rot; GY, Grain yield.

**Table 6:** Ranking of the top 15 and bottom five experimental hybrids under PC1BI, PC2BI, and LD_AV selection schemes.

Hybrids	LD_AV	ED	ER	GY	Value	Rank	Hybrids	LD_AV	ED	ER	GY	Value	Rank	Hybrids	LD_AV	ED	ER	GY	Rank
Top 15 hybrids based on PC1BI							Top 15 hybrids based on PC2BI							Top 15 hybrids based on direct LD_AV					
E154	4.8	1.7	2.5	6.5	-8.7	1	E28	5.1	1.4	1.1	5.0	1.4	1	E163	4.6	1.7	2.5	5.1	1
E163	4.6	1.7	2.5	5.1	-9.3	2	E155	4.8	1.6	1.7	4.1	0.3	2	E152	4.7	1.6	3.8	4.4	2
E133	4.9	2.1	7.2	7.8	-9.4	3	E156	4.9	1.7	2.4	4.5	-0.3	3	E146	4.7	1.6	6.1	4.1	3
E116	4.9	1.7	2.9	6.1	-9.5	4	E154	4.8	1.7	2.5	6.5	-0.3	4	E164	4.7	1.7	3.7	4.3	4
E141	4.7	2.2	7.9	7.6	-9.5	5	E163	4.6	1.7	2.5	5.1	-0.5	5	E165	4.7	1.9	9.2	4.7	5
E78	4.9	1.9	5.6	7.0	-9.6	6	E12	5.3	1.6	3.2	5.3	-0.7	6	E141	4.7	2.2	7.9	7.6	6
E159	5.0	1.9	7.0	7.7	-9.7	7	E161	4.9	1.7	2.9	5.7	-0.7	7	E183	4.7	1.8	5.2	5.7	7
E34	4.9	1.5	4.1	6.2	-9.8	8	E116	4.9	1.7	2.9	6.1	-0.8	8	E169	4.7	2.1	6.6	5.2	8
E148	4.8	1.8	4.1	5.8	-9.8	9	E1	5.2	1.7	3.3	4.7	-1.0	9	E148	4.8	1.8	4.1	5.8	9
E74	5.2	1.6	3.8	6.8	-9.8	10	E172	5.3	1.6	3.5	4.1	-1.0	10	E139	4.8	2.0	7.8	4.7	10
E161	4.9	1.7	2.9	5.7	-9.9	11	E74	5.2	1.6	3.8	6.8	-1.2	11	E185	4.8	1.9	7.3	5.7	11
E115	5.0	1.8	7.0	7.4	-10.0	12	E126	5.1	1.8	3.4	4.0	-1.2	12	E89	4.8	2.2	11.4	4.2	12
E181	4.8	2.1	7.5	7.0	-10.2	13	E18	4.9	1.7	3.5	5.0	-1.3	13	E117	4.8	2.3	12.8	5.7	13
E111	5.1	1.7	5.3	6.6	-10.3	14	E38	5.3	1.7	3.7	3.0	-1.4	14	E170	4.8	2.0	4.6	4.9	14
E183	4.7	1.8	5.2	5.7	-10.3	15	E130	5.2	1.7	3.8	5.9	-1.5	15	E155	4.8	1.6	1.7	4.1	15
Bottom 5 Hybrids							Bottom 5 Hybrids							Bottom 5 Hybrids					
E189	5.6	2.6	20.0	5.1	-19.3	188	E140	5.1	2.5	19.2	5.7	-16.7	188	E41	5.6	2.0	3.9	3.4	188
E188	5.0	2.5	20.5	3.5	-19.3	189	E189	5.6	2.6	20.0	5.1	-17.2	189	E189	5.6	2.6	20	5.1	189
E127	5.0	2.9	23.9	2.8	-21.5	190	E188	5.0	2.5	20.5	3.5	-18.1	190	E2	5.6	1.8	5.9	2.7	190
E192	6.1	2.3	17.4	3.1	-21.7	191	E127	5.0	2.9	23.9	2.8	-21.7	191	E192	6.1	2.3	17.4	3.1	191
E191	6.2	2.7	31.3	4.4	-26.3	192	E191	6.2	2.7	31.3	4.4	-27.6	192	E191	6.2	2.7	31.3	4.4	192

LD1, LD2, LD3, Leaf feeding damage score at 7, 14, 21 days after infestation, respectively; LD_AV, Average leaf feeding damage score; ED, Ear damage; ER, Ear rot; GY, Grain yield.

**Table 7:** Percentage relative selection gain of each of the 15 best-selected hybrids against the commercial checks WH401 and WH505 under different selection schemes.

PC1BI selection index					PC2BI selection index					Direct LD_AV selection scheme				
Against WH401														
Hybrids	LD_AV	ED	ER	GY	Hybrids	LD_AV	ED	ER	GY	Hybrids	LD_AV	ED	ER	GY
E154	-20.3	-26.6	-85.9	112.0	E28	-15.6	-39.9	-93.5	62.1	E163	-24.5	-26.9	-85.9	67.3
E163	-24.5	-26.9	-85.9	67.3	E155	-21.1	-29.7	-90.0	34.9	E152	-23.1	-29.6	-78.1	43.0
E133	-19.7	-9.10	-58.8	154.5	E156	-19.3	-26.1	-86.2	47.2	E146	-22.8	-30.7	-64.9	34.1
E116	-19.3	-23.9	-83.1	98.1	E154	-20.3	-26.6	-85.9	112.0	E164	-22.6	-23.6	-78.7	39.5
E141	-22.2	-4.70	-54.7	147.6	E163	-24.5	-26.9	-85.9	67.3	E165	-22.3	-16.8	-47.5	52.9
E78	-20.0	-18.2	-68.1	127.2	E12	-13.6	-29.8	-81.8	71.6	E141	-22.2	-4.7	-54.7	147.6
E159	-18.1	-16.6	-60.1	152.7	E161	-18.8	-24.1	-83.1	86.6	E183	-22.2	-22.1	-70.0	85.3
E34	-19.0	-33.7	-76.8	102.7	E116	-19.3	-23.9	-83.1	98.1	E169	-22.1	-7.8	-62.3	69.3
E148	-21.9	-21.4	-76.3	88.4	E1	-14.8	-25.5	-81.4	54.4	E148	-21.9	-21.4	-76.3	88.4
E74	-15.2	-30.5	-78.1	121.4	E172	-13.0	-31.6	-79.9	35.5	E139	-21.7	-14.1	-55.5	53.6
E161	-18.8	-24.1	-83.1	86.6	E74	-15.2	-30.5	-78.1	121.4	E185	-21.6	-15.3	-58.4	84.8
E115	-18.1	-22.6	-59.7	140.6	E126	-16.4	-21.9	-80.3	30.9	E89	-21.5	-5.3	-34.4	38.3
E181	-20.6	-5.8	-56.9	127.6	E18	-18.6	-23.7	-80.2	64.6	E117	-21.4	-0.4	-26.5	86.5
E111	-16.7	-25.6	-69.6	117.2	E38	-12.5	-24.1	-78.7	-1.8	E170	-21.4	-13.3	-73.6	60.3
E183	-22.2	-22.1	-70.0	85.3	E130	-15.0	-23.0	-78.5	91.3	E155	-21.1	-29.7	-90.0	34.9
Against WH501														
E154	-22.4	-38.9	-92.1	47.1	E28	-17.8	-50.0	-96.4	12.4	E163	-26.4	-39.2	-92.1	16
E163	-26.4	-39.2	-92.1	16.0	E155	-23.1	-41.5	-94.4	-6.4	E152	-25.1	-41.5	-87.8	-0.8
E133	-21.8	-24.4	-77.0	76.6	E156	-21.4	-38.5	-92.3	2.1	E146	-24.8	-42.3	-80.4	-7.0
E116	-21.4	-36.7	-90.6	37.4	E154	-22.4	-38.9	-92.1	47.1	E164	-24.7	-36.4	-88.1	-3.2
E141	-24.2	-20.8	-74.7	71.8	E163	-26.4	-39.2	-92.1	16.0	E165	-24.3	-30.8	-70.7	6.1
E78	-22.0	-31.9	-82.2	57.6	E12	-15.8	-41.6	-89.8	19.0	E141	-24.2	-20.8	-74.7	71.8
E159	-20.2	-30.6	-77.8	75.3	E161	-20.9	-36.9	-90.6	29.5	E183	-24.2	-35.2	-83.3	28.6
E34	-21.2	-44.8	-87.1	40.6	E116	-21.4	-36.7	-90.6	37.4	E169	-24.1	-23.3	-79.0	17.4
E148	-23.9	-34.6	-86.8	30.7	E1	-17.1	-38.0	-89.6	7.1	E148	-23.9	-34.6	-86.8	30.7
E74	-17.4	-42.2	-87.8	53.6	E172	-15.2	-43.1	-88.8	-6.0	E139	-23.7	-28.5	-75.2	6.6
E161	-20.9	-36.9	-90.6	29.5	E74	-17.4	-42.2	-87.8	53.6	E185	-23.6	-29.6	-76.8	28.2
E115	-20.3	-35.6	-77.5	66.9	E126	-18.6	-35.0	-89.0	-9.2	E89	-23.5	-21.3	-63.5	-4.1
E181	-22.7	-21.6	-76.0	57.9	E18	-20.8	-36.6	-89.0	14.2	E117	-23.4	-17.2	-59.0	29.4
E111	-18.9	-38.1	-83.1	50.7	E38	-14.8	-36.9	-88.1	-31.9	E170	-23.4	-27.9	-85.3	11.2
E183	-24.2	-35.2	-83.3	28.6	E130	-17.2	-35.9	-88.0	32.7	E155	-23.1	-41.5	-94.4	-6.4

LD1, LD2, LD3, Leaf feeding damage score at 7, 14, 21 days after infestation, respectively; ED, Ear damage; ER, Ear rot; GY, Grain yield; PC1BI, Principal component 1-based index; PC2BI, Principal component 2-based index.

## Discussion

Until 2016, when FAW was reported in Africa (Goergen et al., 2016), it had primarily been a maize pest in the Americas. Its emergence on the African continent prompted the efforts to identify suitable sources of genetic resistance within the available germplasm to develop resistant maize hybrids. These initial breeding efforts, conducted under artificial FAW infestation, led to the identification of several inbred lines with tolerance to FAW (Prasanna et al., 2022). To develop suitable hybrids, these inbred lines must be evaluated in hybrid combination under different management conditions. This process requires consideration of numerous traits to identify the most suitable genotypes. Given the large number of traits to be considered in a breeding strategy, selection of the most relevant ones can be challenging. This process thus requires an efficient approach for selecting hybrids that combine resistance to FAW, good agronomic performance, and a number of essential adaptive traits. Therefore, this study explored the use of principal component-based selection indices to efficiently identify genotypes that combine the desired traits under artificial FAW infestation. The key traits important for FAW resistance breeding in the study included leaf feeding damage, ear damage, ear rot incidence, and grain yield.

### Genetic variation and heritability estimates

Significant genotypic variation was observed for all FAW resistance parameters and agronomic traits, indicating substantial genetic variability in this germplasm for breeding for host resistance to FAW. Genetic variability is critical to making genetic progress in breeding. The findings are in agreement with previous investigations on FAW that reported genetic variations for FAW resistance related traits in tropical and temperate maize (Kasoma et al., 2021; Kamweru et al., 2022; Soujanya et al., 2022; Warburton et al., 2023). The development of the insect-resistant populations that are progenitors of some of the lines used in this study was based on a diverse set of Caribbean maize and Tuxpeño accessions from Mexico (Mihm, 1997). This underscores the importance of the large genetic variability in addressing breeding for a pest like FAW in a new environment.

In the present study, the environment and G × E interaction were significant, suggesting that the different seasons of evaluation could have led to differential genotype responses. Similar results were reported in earlier studies on FAW (Widstrom et al., 1992; Kasoma et al., 2021; Kamweru et al., 2022; 2023). Differences in temperature over testing seasons are known to influence the biological activities of insects including FAW (Ramirez-Cabral et al., 2017; Schneider et al., 2022; Skendžić et al., 2021; Yan et al., 2022). In plant breeding programs, traits with high heritability are more likely to be improved rapidly (Nyquist, 1991). This study revealed that broad-sense heritability estimates for the FAW resistance parameters were moderate to high, which suggests that breeding for FAW resistance using this germplasm could lead to reasonable genetic gains from selection for these traits. The high broad-sense heritability estimates also suggest that narrow-sense heritability for these FAW-related parameters would be moderate to high (Falconer and Mackay, 1996). Previous studies on FAW have also reported moderate to high broad-sense heritability estimates for FAW resistance parameters in maize (Kasoma et al., 2021; Kamweru et al., 2022; 2023).

### Performance of PC-based indices and hybrid selection

A selection index offers a criterion for selection that objectively assesses the genotypic values of individuals or families (Subandi et al., 1973). An effective selection index requires strong genetic correlations among the traits that are included in an index (Oloyede-Kamiyo, 2019; Olivoto and Nardino, 2021; Batista et al., 2021). In this study, the genotypic correlations among the FAW resistance traits were strong, justifying their inclusion in the selection index. Similarly, strong genotypic correlations between the leaf-feeding damage traits, ED, and ER were reported by Kamweru et al. (2022). One of the challenges of using selection indices is the complexity of assigning economic weights (e.g. Smith, 1936; Smith et al., 1981). Several authors have suggested several economic weight-free indices (Williams, 1962; Subandi et al., 1973; Stromberg and Compton, 1989; Tang et al., 2021). Two economic weight-free PC-based selection indices were developed in this study, which together explained 97% of the variation in this germplasm. The first index, PC1BI explained largely the variation in the three leaf feeding damage traits and GY, whereas PC2BI explained the variation in ear-related traits ED and ER. When both indices were used to select the top 15 hybrids, the checks were not among the top hybrids selected. This was because the commercial check hybrids WH401 and WH505 performed poorly in relation to the key traits for FAW resistance with high loadings on both indices, but it also points to the good discriminatory power of the two indices developed. Tang et al. (2021) demonstrated that using multivariate selection tools, such as PCA, is an effective method for combining multiple traits into a single maize genotype.

The use of PC1BI to select the top 10 or 15 hybrids resulted in balanced gains in the desired direction for all traits, with the largest genetic gain for GY of 19.9% (top 10) and 18.8% for top 15%. Interestingly, the improvements in GY achieved using PC1BI were nearly identical to those obtained by directly selecting for GY. Oloyede-Kamiyo (2019) also reported favorable selection gains for GY in a study evaluating the efficiency of four index-based selection methods for resistance to the lepidopteran stem borer *Chilo partellus*. Several other studies have highlighted the beneficial application of selection indices to identify high-yielding hybrids with favorable trait combinations in maize (Widstrom et al., 1982; Mhike et al., 2012; Makumbi et al., 2018; Crispim-Filho et al., 2020). In contrast, a study on sorghum using the functional plant loss index (FPLI) which is akin to PC1BI, did not show a significant relationship with grain yield performance (Singh et al., 2011). With the application of PC2BI, selection gains in the desired direction were achieved for all traits when selecting the top 10 hybrids, although larger gains were observed for ED and ER. While both indices resulted in desirable gains, PC1BI proved to be a better index, with a significantly higher gain for GY (19.90) compared to 1.68 for PC2BI. Direct selection methods for leaf feeding damage, ear damage or GY, resulted in less balanced selection gains for all traits compared to the PC-based indices. This result further underscores the importance of using selection indices in breeding programs focused on maximizing high yield potential and improving tolerance to multiple stresses.

With the application of the PC1BI index, several promising hybrids with grain yield ≥ 7.0 t ha^-1^ and good overall index scores were identified. These hybrids align with the current priority trait metrics for FAW resistance breeding at CIMMYT (Prasanna et al., 2022). This suggests that PC1BI could serve as an excellent selection index tool in FAW resistance breeding to identify genotypes that combined FAW resistance with high yield potential. Similar recommendations for using selection indices have been made for corn ear worm (Widstrom et al., 1982) and *Chilo partellus* resistance in maize (Oloyede-Kamiyo (2019) and sorghum (Singh et al., 2011). Some hybrids selected using the PC2BI index showed reduced ear and rot damage though their yield was slightly below the 7tha^-1^ threshold. These genotypes warrant further testing as they may carry additional traits beneficial for a breeding program (Ambrósio et al., 2024). The combined selection performance of the two indices (PC1BI and PC2BI), highlighted overlapping selections among the top 15 hybrids. Several of these hybrids were also selected through a direct selection scheme based on LD_AV. Hybrid E163 emerged as a promising genotype, as it was selected across all the selection schemes. The promising genotypes are likely to have a higher frequency of stable and adaptable alleles for both GY and FAW resistance which could be valuable for breeding purposes. Genotypes commonly selected across indices exhibit extensive adaptability alleles and perform well across a variety of environments (Zuffo et al., 2020; Ambrósio et al., 2024). This study was carried out under controlled environments, which excluded other traits important for selection of adapted high yielding maize hybrids, for example response to foliar diseases. Additional field studies across a variety of stress conditions often encountered by a large proportion of smallholder farmers are required to validate the yield stability and disease resistance of the selected hybrids for broader adaptation.

### Potential future application of PC-based indices

Presently, there are no published reports on the implementation of selection indices to select FAW resistant genotypes in tropical maize in SSA where the pest is relatively new. The PCA approach used in this study offers a framework for the construction and use of an economic weight-free multi-trait selection index for the identification of FAW-resistant genotypes in SSA. This would overcome the limits paused by the need to determine economic weights which can limit gains from selection. However, given the multiple stresses present in SSA (e.g. Makumbi et al., 2018), the development of multiple stress-tolerant varieties suitable for farmers requires selection for a wide range of traits. Therefore, applying a PC-based selection index that includes other important traits in SSA is crucial for optimizing selection gains across multiple traits. Such traits include but are not limited to agronomic traits like lodging resistance, plant height, ear rot, poor husk cover, fungal and viral diseases. Incorporating many traits will help the validation of the PC-based indices as a handy tool for breeders to select for multiple traits. A further area of study could be the incorporation of farmer responses in the PC-based indices. Data collection on a large number of agronomic and disease traits is time consuming and errors can be introduced. We propose integrating advancement in technology during data collection as breeders validate and test indices. For instance, high-throughput phenotyping methods (e.g., Araus et al., 2018) could be used to effectively collect real-time and high-quality data on FAW leaf feeding and ear damage, which are typically costly and labor-intensive traits to measure at multiples intervals. Use of high-throughput phenotyping methods would help to reduce labor costs for breeding programs while increasing efficiency and precision in data collection. Similar high-throughput approaches could be implemented for other agronomic traits like plant and ear height, ear and kernel traits, foliar leaf diseases, and virus diseases like maize lethal necrosis and maize streak virus to further reduce the cost of breeding operations. With reduction in cost, the resources can then be allocated to other breeding operations like acquisition of electronic data collection equipment that improve efficiency and drive genetic gain.

## Conclusions

Two principal component (PC)-based indices were developed and evaluated for their effectiveness in selection of FAW-resistant hybrids and achieving desired gains in FAW resistance breeding. Both PC indices explained most of the variation among the genotypes, with PC1BI accounting for most of the variation for FAW leaf feeding damage and grain yield. Larger desired gains were achieved using PC1BI. The PC-based indices resulted in more balanced multi-trait genetic gains compared to the single-trait selection approach. The use of PC-based index selection presents a promising, economic weight-free selection tool to maximize genetic gains in FAW resistance breeding programs. However, it should also incorporate additional desirable agronomic and adaptive traits beyond the FAW-related parameters.

## Data Availability

The data sets used in this study have all been analyzed and summarized in the main Tables, Figures, and Supplementary Tables presented.

## Supplementary material

Supplementary Figure 1 Scree plot showing eigenvalues for the seven PCs.
