# Supplementary figures and images for: Use of multi-trait principal component selection index to identify fall armyworm (Spodoptera frugiperda) resistant maize genotypes
Source: Front Plant Sci. 2025 Mar 27;16:1544010. doi: 10.3389/fpls.2025.1544010 (PMC11983501; doi:10.3389/fpls.2025.1544010)

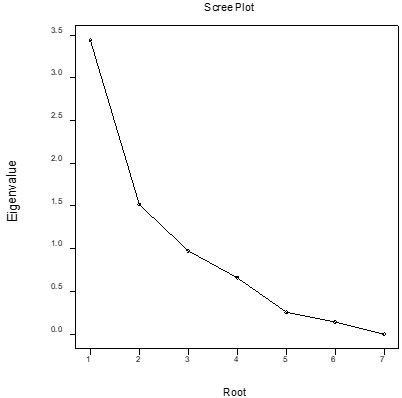

Supplement: Supplementary Figure 1 — Scree plot showing eigenvalues for the seven PCs. [file Image1.jpeg]
