# Supplementary material for: Use of multi-trait principal component selection index to identify fall armyworm (Spodoptera frugiperda) resistant maize genotypes
Source: Front Plant Sci. 2025 Mar 27;16:1544010. doi: 10.3389/fpls.2025.1544010 (PMC11983501; doi:10.3389/fpls.2025.1544010)
Supplement: Supplementary file 2 [file Table1.docx]

Supplementary Table 1: Mean for FAW resistance parameters and index score values of 192 hybrids evaluated in four environments in 2020-2022.

| Hybrid codes | Hybrid names | LD1 | LD2 | LD3 | LD_AV | ED | ER | GY | PC1BI | PC2BI |
| --- | --- | --- | --- | --- | --- | --- | --- | --- | --- | --- |
| E1 | CML247/CML269 | 3.7 | 6.0 | 5.8 | 5.2 | 1.7 | 3.3 | 4.7 | -11.7 | -1.0 |
| E2 | CML247/CML17 | 3.9 | 6.5 | 6.5 | 5.6 | 1.8 | 5.9 | 2.7 | -16.1 | -3.3 |
| E3 | CML247/CML23 | 3.8 | 6.3 | 6.1 | 5.4 | 2.2 | 11.0 | 3.9 | -16.3 | -8.6 |
| E4 | CML247/CML274 | 3.4 | 6.1 | 5.8 | 5.1 | 1.7 | 4.2 | 5.9 | -10.7 | -1.8 |
| E5 | CML247/CML371 | 3.8 | 6.2 | 5.8 | 5.3 | 2.4 | 12.9 | 4.2 | -16.5 | -10.6 |
| E6 | CML247/CML372 | 3.6 | 6.4 | 6.1 | 5.4 | 2.1 | 10.4 | 4.7 | -15.1 | -7.8 |
| E7 | CML247/CML402 | 3.9 | 6.3 | 5.8 | 5.4 | 2.2 | 12.7 | 3.8 | -17.0 | -10.2 |
| E8 | CML247/CML476 | 3.7 | 6.2 | 5.7 | 5.2 | 2.1 | 8.3 | 3.4 | -15.2 | -6.1 |
| E9 | CML247/DL187008 | 3.8 | 6.2 | 6.0 | 5.3 | 1.9 | 6.6 | 3.9 | -14.3 | -4.3 |
| E10 | CML247/DL187009 | 3.6 | 6.4 | 5.6 | 5.2 | 2.4 | 12.0 | 4.9 | -15.2 | -9.8 |
| E11 | CML247/DL187010 | 3.6 | 6.1 | 5.8 | 5.2 | 1.8 | 4.8 | 4.7 | -12.3 | -2.4 |
| E12 | CML247/DL187011 | 3.7 | 6.3 | 5.8 | 5.3 | 1.6 | 3.2 | 5.3 | -11.4 | -0.7 |
| E13 | CML247/DL187012 | 3.8 | 6.0 | 5.9 | 5.2 | 1.8 | 5.0 | 3.7 | -13.6 | -2.7 |
| E14 | CML247/DL187019 | 3.3 | 6.0 | 5.8 | 5.1 | 1.9 | 6.4 | 3.8 | -13.6 | -4.2 |
| E15 | CML247/CML334 | 3.8 | 6.3 | 6.1 | 5.4 | 2.4 | 15.5 | 2.7 | -19.3 | -13.0 |
| E16 | CML247/CKSBL10153 | 3.7 | 6.3 | 6.0 | 5.4 | 1.8 | 6.6 | 5.0 | -13.3 | -4.1 |
| E17 | CML247/CML71 | 3.6 | 6.1 | 5.6 | 5.1 | 2.1 | 6.0 | 4.0 | -13.5 | -4.0 |
| E18 | CML247/CML370 | 3.4 | 6.0 | 5.4 | 4.9 | 1.7 | 3.5 | 5.0 | -10.8 | -1.3 |
| E19 | CML269/CML17 | 3.6 | 6.3 | 6.0 | 5.3 | 1.9 | 4.8 | 4.7 | -12.8 | -2.4 |
| E20 | CML269/CML23 | 3.5 | 6.0 | 6.1 | 5.2 | 1.7 | 6.7 | 4.1 | -13.8 | -4.2 |
| E21 | CML269/CML274 | 3.6 | 6.4 | 6.1 | 5.3 | 1.6 | 4.4 | 5.8 | -11.6 | -1.7 |
| E22 | CML269/CML371 | 3.4 | 6.2 | 5.8 | 5.1 | 2.1 | 12.4 | 3.2 | -16.7 | -10.0 |
| E23 | CML269/CML372 | 3.5 | 6.0 | 6.0 | 5.2 | 1.8 | 7.2 | 5.3 | -12.6 | -4.6 |
| E24 | CML269/CML402 | 3.6 | 6.1 | 5.5 | 5.1 | 1.6 | 4.1 | 5.4 | -11.1 | -1.7 |
| E25 | CML269/CML476 | 3.7 | 6.1 | 5.9 | 5.2 | 1.5 | 5.4 | 4.5 | -12.9 | -2.7 |
| E26 | CML269/DL187008 | 3.5 | 6.0 | 5.6 | 5.0 | 1.8 | 7.0 | 6.3 | -11.2 | -4.6 |
| E27 | CML269/DL187009 | 3.6 | 6.1 | 5.8 | 5.2 | 1.9 | 7.1 | 5.5 | -12.5 | -4.8 |
| E28 | CML269/DL187010 | 3.5 | 5.9 | 5.9 | 5.1 | 1.4 | 1.1 | 5.0 | -10.5 | 1.4 |
| E29 | CML269/DL187011 | 3.4 | 5.9 | 5.4 | 4.9 | 1.5 | 3.9 | 5.0 | -10.9 | -1.5 |
| E30 | CML269/DL187012 | 3.3 | 5.9 | 5.5 | 4.9 | 1.9 | 9.5 | 5.7 | -12.5 | -7.1 |
| E31 | CML269/DL187019 | 3.2 | 5.8 | 5.6 | 4.9 | 1.6 | 4.2 | 4.3 | -11.6 | -1.9 |
| E32 | CML269/CML334 | 3.7 | 6.1 | 5.6 | 5.1 | 1.8 | 8.7 | 5.8 | -12.6 | -6.2 |
| E33 | CML269/CKSBL10153 | 3.4 | 6.0 | 5.9 | 5.1 | 1.7 | 6.3 | 5.6 | -11.8 | -3.8 |
| E34 | CML269/CML71 | 3.3 | 5.9 | 5.6 | 4.9 | 1.5 | 4.1 | 6.2 | -9.8 | -1.6 |
| E35 | CML269/CML370 | 3.3 | 5.8 | 5.5 | 4.9 | 1.8 | 9.0 | 4.8 | -13.0 | -6.6 |
| E36 | CML17/CML23 | 3.6 | 6.2 | 6.2 | 5.3 | 2.0 | 5.1 | 5.6 | -12.0 | -2.7 |
| E37 | CML17/CML274 | 3.8 | 6.5 | 6.3 | 5.5 | 2.0 | 10.6 | 2.9 | -17.5 | -8.0 |
| E38 | CML17/CML371 | 3.8 | 6.1 | 6.0 | 5.3 | 1.7 | 3.7 | 3.0 | -14.0 | -1.4 |
| E39 | CML17/CML372 | 3.5 | 6.2 | 5.9 | 5.2 | 1.9 | 6.7 | 5.5 | -12.4 | -4.3 |
| E40 | CML17/CML402 | 4.0 | 6.2 | 6.0 | 5.4 | 2.0 | 9.0 | 2.5 | -16.9 | -6.6 |
| E41 | CML17/CML476 | 4.0 | 6.4 | 6.3 | 5.6 | 2.0 | 3.9 | 3.4 | -14.4 | -1.7 |
| E42 | CML17/DL187008 | 3.6 | 6.2 | 6.1 | 5.3 | 1.9 | 5.9 | 4.8 | -13.0 | -3.5 |
| E43 | CML17/DL187009 | 3.6 | 6.3 | 5.9 | 5.3 | 2.3 | 12.5 | 5.1 | -15.3 | -10.1 |
| E44 | CML17/DL187010 | 3.7 | 6.0 | 5.6 | 5.1 | 1.7 | 3.9 | 4.6 | -11.9 | -1.7 |
| E45 | CML17/DL187011 | 3.7 | 6.5 | 6.0 | 5.4 | 1.7 | 4.9 | 4.3 | -13.5 | -2.4 |
| E46 | CML17/DL187012 | 3.7 | 6.3 | 6.2 | 5.4 | 2.2 | 14.1 | 4.9 | -16.5 | -11.4 |
| E47 | CML17/DL187019 | 3.6 | 6.1 | 5.7 | 5.2 | 1.8 | 4.6 | 4.0 | -13.0 | -2.4 |
| E48 | CML17/CML334 | 3.9 | 6.0 | 5.9 | 5.3 | 2.1 | 14.3 | 5.0 | -16.2 | -11.6 |
| E49 | CML17/CKSBL10153 | 3.8 | 6.0 | 5.8 | 5.2 | 1.8 | 5.0 | 6.2 | -11.0 | -2.7 |
| E50 | CML17/CML71 | 3.4 | 6.2 | 6.0 | 5.2 | 2.0 | 8.4 | 4.2 | -14.3 | -6.1 |
| E51 | CML17/CML370 | 3.3 | 5.9 | 5.4 | 4.9 | 1.8 | 3.5 | 5.1 | -10.6 | -1.6 |
| E52 | CML23/CML274 | 3.6 | 6.3 | 6.0 | 5.3 | 2.2 | 10.4 | 3.1 | -16.7 | -8.2 |
| E53 | CML23/CML371 | 3.5 | 6.2 | 6.1 | 5.2 | 2.3 | 14.8 | 3.4 | -17.9 | -12.4 |
| E54 | CML23/CML372 | 3.4 | 6.3 | 6.4 | 5.3 | 1.9 | 7.9 | 4.7 | -14.1 | -5.4 |
| E55 | CML23/CML402 | 3.4 | 6.3 | 6.0 | 5.2 | 2.0 | 14.1 | 5.5 | -15.5 | -11.3 |
| E56 | CML23/CML476 | 3.6 | 6.2 | 6.1 | 5.3 | 2.4 | 17.3 | 4.7 | -17.7 | -14.8 |
| E57 | CML23/DL187008 | 3.6 | 6.2 | 6.0 | 5.2 | 2.3 | 12.3 | 5.9 | -14.4 | -9.9 |
| E58 | CML23/DL187009 | 3.7 | 6.1 | 6.3 | 5.4 | 2.6 | 14.4 | 5.7 | -15.9 | -12.2 |
| E59 | CML23/DL187010 | 3.2 | 6.1 | 5.9 | 5.1 | 2.0 | 8.3 | 5.4 | -12.8 | -5.9 |
| E60 | CML23/DL187011 | 3.3 | 6.1 | 5.9 | 5.1 | 1.9 | 7.1 | 6.2 | -11.6 | -4.7 |
| E61 | CML23/DL187012 | 3.4 | 6.2 | 6.2 | 5.3 | 1.7 | 6.3 | 5.9 | -12.0 | -3.6 |
| E62 | CML23/DL187019 | 3.4 | 6.2 | 5.9 | 5.1 | 1.7 | 4.8 | 5.6 | -11.4 | -2.3 |
| E63 | CML23/CML334 | 3.4 | 6.2 | 6.1 | 5.2 | 2.1 | 9.5 | 5.7 | -13.4 | -7.1 |
| E64 | CML23/CKSBL10153 | 3.5 | 6.1 | 5.9 | 5.1 | 2.3 | 10.9 | 5.9 | -13.6 | -8.7 |
| E65 | CML23/CML71 | 3.2 | 6.0 | 5.8 | 5.0 | 2.3 | 11.9 | 3.4 | -16.1 | -9.8 |
| E66 | CML23/CML370 | 3.1 | 6.1 | 6.0 | 5.1 | 2.0 | 7.0 | 6.0 | -11.6 | -4.7 |
| E67 | CML274/CML371 | 3.5 | 6.2 | 5.9 | 5.2 | 2.0 | 9.5 | 4.5 | -14.5 | -7.1 |
| E68 | CML274/CML372 | 3.7 | 6.4 | 6.2 | 5.4 | 2.1 | 7.9 | 3.7 | -15.3 | -5.5 |
| E69 | CML274/CML402 | 3.7 | 6.3 | 5.7 | 5.2 | 2.2 | 16.6 | 4.6 | -17.4 | -13.9 |
| E70 | CML274/CML476 | 3.5 | 6.1 | 5.4 | 5.0 | 2.2 | 9.5 | 4.2 | -14.3 | -7.4 |
| E71 | CML274/DL187008 | 3.7 | 6.2 | 5.7 | 5.2 | 2.3 | 11.8 | 7.6 | -12.3 | -9.4 |
| E72 | CML274/DL187009 | 3.8 | 6.3 | 5.9 | 5.3 | 2.4 | 17.1 | 6.5 | -16.0 | -14.5 |
| E73 | CML274/DL187010 | 3.6 | 6.3 | 5.9 | 5.3 | 1.9 | 4.6 | 4.7 | -12.7 | -2.4 |
| E74 | CML274/DL187011 | 3.4 | 6.4 | 5.7 | 5.2 | 1.6 | 3.8 | 6.8 | -9.8 | -1.2 |
| E75 | CML274/DL187012 | 3.8 | 6.3 | 5.8 | 5.3 | 1.6 | 4.4 | 6.5 | -10.7 | -1.7 |
| E76 | CML274/DL187019 | 3.5 | 6.1 | 5.9 | 5.1 | 1.7 | 4.4 | 6.0 | -10.8 | -2.0 |
| E77 | CML274/CML334 | 3.8 | 6.5 | 6.2 | 5.5 | 2.3 | 11.9 | 5.8 | -15.0 | -9.3 |
| E78 | CML274/CKSBL10153 | 3.5 | 5.8 | 5.2 | 4.9 | 1.9 | 5.6 | 7.0 | -9.6 | -3.4 |
| E79 | CML274/CML71 | 3.3 | 6.2 | 5.9 | 5.1 | 2.0 | 3.5 | 5.4 | -11.2 | -1.5 |
| E80 | CML274/CML370 | 3.5 | 6.3 | 5.8 | 5.2 | 2.0 | 7.9 | 6.0 | -12.3 | -5.5 |
| E81 | CML371/CML372 | 3.7 | 6.1 | 6.0 | 5.2 | 2.1 | 10.0 | 4.4 | -14.9 | -7.6 |
| E82 | CML371/CML402 | 3.8 | 5.9 | 5.5 | 5.1 | 2.4 | 15.2 | 5.7 | -15.4 | -12.9 |
| E83 | CML371/CML476 | 3.4 | 5.9 | 5.6 | 5.0 | 2.3 | 12.8 | 4.0 | -15.8 | -10.6 |
| E84 | CML371/DL187008 | 3.7 | 6.2 | 6.0 | 5.3 | 2.2 | 9.2 | 4.3 | -15.0 | -6.9 |
| E85 | CML371/DL187009 | 3.7 | 5.7 | 5.3 | 4.9 | 2.2 | 10.9 | 6.1 | -12.6 | -8.8 |
| E86 | CML371/DL187010 | 3.4 | 5.9 | 5.7 | 5.0 | 1.9 | 7.3 | 3.7 | -13.9 | -5.1 |
| E87 | CML371/DL187011 | 3.2 | 6.0 | 5.5 | 4.9 | 1.7 | 4.9 | 5.3 | -11.0 | -2.6 |
| E88 | CML371/DL187012 | 3.5 | 6.0 | 5.7 | 5.1 | 1.9 | 8.3 | 5.2 | -13.0 | -5.9 |
| E89 | CML371/DL187019 | 3.2 | 5.6 | 5.5 | 4.8 | 2.2 | 11.4 | 4.2 | -14.4 | -9.3 |
| E90 | CML371/CML334 | 3.4 | 5.8 | 5.7 | 4.9 | 2.0 | 8.6 | 5.0 | -12.9 | -6.3 |
| E91 | CML371/CKSBL10153 | 3.3 | 5.7 | 5.5 | 4.9 | 2.0 | 4.7 | 4.9 | -11.3 | -2.8 |
| E92 | CML371/CML71 | 3.4 | 6.0 | 5.5 | 4.9 | 1.9 | 6.3 | 3.9 | -13.1 | -4.1 |
| E93 | CML371/CML370 | 3.1 | 5.8 | 5.8 | 4.9 | 2.1 | 6.6 | 4.2 | -12.9 | -4.6 |
| E94 | CML372/CML402 | 3.8 | 6.3 | 5.9 | 5.3 | 1.9 | 7.9 | 5.5 | -13.2 | -5.3 |
| E95 | CML372/CML476 | 3.4 | 6.2 | 5.9 | 5.2 | 1.8 | 5.8 | 4.8 | -12.7 | -3.4 |
| E96 | CML372/DL187008 | 3.6 | 6.3 | 5.7 | 5.2 | 2.0 | 7.1 | 7.2 | -10.8 | -4.8 |
| E97 | CML372/DL187009 | 3.6 | 6.0 | 5.9 | 5.2 | 2.6 | 17.7 | 7.1 | -15.2 | -15.2 |
| E98 | CML372/DL187010 | 3.7 | 6.0 | 6.0 | 5.3 | 1.9 | 5.9 | 5.5 | -12.3 | -3.5 |
| E99 | CML372/DL187011 | 3.5 | 5.9 | 6.1 | 5.1 | 1.8 | 9.4 | 5.3 | -13.4 | -6.8 |
| E100 | CML372/DL187012 | 3.5 | 6.1 | 5.8 | 5.1 | 1.9 | 13.2 | 5.9 | -14.3 | -10.3 |
| E101 | CML372/DL187019 | 3.4 | 5.9 | 5.8 | 5.0 | 1.9 | 5.0 | 6.0 | -10.6 | -2.7 |
| E102 | CML372/CML334 | 3.6 | 6.3 | 6.2 | 5.4 | 2.0 | 8.1 | 6.6 | -12.4 | -5.6 |
| E103 | CML372/CKSBL10153 | 3.3 | 6.0 | 5.8 | 5.0 | 2.0 | 8.2 | 7.7 | -10.4 | -5.8 |
| E104 | CML372/CML71 | 3.2 | 6.0 | 6.1 | 5.1 | 1.7 | 5.1 | 5.5 | -11.5 | -2.7 |
| E105 | CML372/CML370 | 3.6 | 6.0 | 6.1 | 5.2 | 1.9 | 7.0 | 4.4 | -13.7 | -4.5 |
| E106 | CML402/CML476 | 3.5 | 6.2 | 5.5 | 5.1 | 2.0 | 9.1 | 6.6 | -12.0 | -6.7 |
| E107 | CML402/DL187008 | 3.4 | 6.0 | 5.7 | 5.0 | 2.0 | 8.3 | 7.2 | -10.8 | -6.0 |
| E108 | CML402/DL187009 | 3.6 | 6.1 | 5.9 | 5.2 | 2.3 | 11.7 | 6.4 | -13.5 | -9.4 |
| E109 | CML402/DL187010 | 3.7 | 6.0 | 5.4 | 5.0 | 2.3 | 10.9 | 6.2 | -13.0 | -8.8 |
| E110 | CML402/DL187011 | 3.6 | 6.3 | 5.6 | 5.2 | 1.9 | 8.2 | 6.6 | -11.8 | -5.7 |
| E111 | CML402/DL187012 | 3.5 | 6.0 | 5.8 | 5.1 | 1.7 | 5.3 | 6.6 | -10.3 | -2.8 |
| E112 | CML402/DL187019 | 3.4 | 6.0 | 5.1 | 4.9 | 2.1 | 8.8 | 6.1 | -11.7 | -6.7 |
| E113 | CML402/CML334 | 3.5 | 6.2 | 5.6 | 5.1 | 2.4 | 13.0 | 6.7 | -13.5 | -10.7 |
| E114 | CML402/CKSBL10153 | 3.7 | 6.0 | 5.4 | 5.0 | 2.1 | 10.3 | 6.9 | -12.1 | -8.0 |
| E115 | CML402/CML71 | 3.5 | 5.8 | 5.6 | 5.0 | 1.8 | 7.0 | 7.4 | -10.0 | -4.6 |
| E116 | CML402/CML370 | 3.3 | 5.9 | 5.5 | 4.9 | 1.7 | 2.9 | 6.1 | -9.5 | -0.8 |
| E117 | CML476/DL187008 | 3.2 | 5.9 | 5.2 | 4.8 | 2.3 | 12.8 | 5.7 | -13.5 | -10.7 |
| E118 | CML476/DL187009 | 3.6 | 5.8 | 5.5 | 5.0 | 2.2 | 8.6 | 3.9 | -14.1 | -6.6 |
| E119 | CML476/DL187010 | 3.5 | 5.9 | 5.9 | 5.1 | 2.4 | 9.5 | 4.5 | -14.4 | -7.6 |
| E120 | CML476/DL187011 | 3.6 | 5.7 | 5.5 | 4.9 | 1.8 | 4.8 | 5.9 | -10.5 | -2.6 |
| E121 | CML476/DL187012 | 3.9 | 6.0 | 5.5 | 5.1 | 1.9 | 6.9 | 4.9 | -12.9 | -4.6 |
| E122 | CML476/DL187019 | 3.7 | 6.0 | 5.4 | 5.0 | 2.0 | 10.6 | 3.0 | -16.0 | -8.3 |
| E123 | CML476/CML334 | 3.4 | 6.1 | 5.8 | 5.1 | 2.4 | 15.5 | 5.7 | -15.4 | -13.1 |
| E124 | CML476/CKSBL10153 | 4.1 | 6.2 | 5.2 | 5.2 | 2.0 | 7.9 | 2.2 | -16.2 | -5.8 |
| E125 | CML476/CML71 | 3.5 | 5.7 | 5.4 | 4.8 | 1.8 | 5.6 | 4.1 | -12.4 | -3.6 |
| E126 | CML476/CML370 | 3.3 | 5.9 | 6.0 | 5.1 | 1.8 | 3.4 | 4.0 | -12.3 | -1.2 |
| E127 | DL187008/DL187009 | 3.6 | 6.1 | 5.3 | 5.0 | 2.9 | 23.9 | 2.8 | -21.5 | -21.7 |
| E128 | DL187008/DL187010 | 3.4 | 6.0 | 5.5 | 5.0 | 1.9 | 6.7 | 6.5 | -10.8 | -4.5 |
| E129 | DL187008/DL187011 | 3.3 | 5.9 | 5.5 | 4.9 | 1.8 | 9.1 | 6.7 | -11.3 | -6.6 |
| E130 | DL187008/DL187012 | 3.9 | 6.1 | 5.5 | 5.2 | 1.7 | 3.8 | 5.9 | -10.8 | -1.5 |
| E131 | DL187008/DL187019 | 3.4 | 5.6 | 5.5 | 4.8 | 1.9 | 7.9 | 5.4 | -11.9 | -5.6 |
| E132 | DL187008/CML334 | 3.8 | 6.1 | 5.9 | 5.3 | 2.2 | 10.4 | 6.2 | -13.3 | -8.0 |
| E133 | DL187008/CKSBL10153 | 3.4 | 5.9 | 5.3 | 4.9 | 2.1 | 7.2 | 7.8 | -9.4 | -5.1 |
| E134 | DL187008/CML71 | 3.7 | 5.5 | 5.3 | 4.8 | 1.9 | 7.7 | 5.8 | -11.4 | -5.6 |
| E135 | DL187008/CML370 | 3.3 | 5.8 | 5.7 | 4.9 | 2.1 | 6.5 | 4.7 | -12.4 | -4.5 |
| E136 | DL187009/DL187010 | 3.3 | 5.9 | 5.7 | 5.0 | 1.9 | 7.3 | 6.2 | -11.3 | -4.9 |
| E137 | DL187009/DL187011 | 3.5 | 5.8 | 5.8 | 5.0 | 1.9 | 6.4 | 6.2 | -11.0 | -4.1 |
| E138 | DL187009/DL187012 | 3.6 | 5.8 | 5.7 | 5.0 | 2.1 | 10.9 | 4.8 | -14.3 | -8.6 |
| E139 | DL187009/DL187019 | 3.3 | 5.5 | 5.6 | 4.8 | 2.0 | 7.8 | 4.7 | -12.4 | -5.7 |
| E140 | DL187009/CML334 | 3.7 | 5.9 | 5.6 | 5.1 | 2.5 | 19.2 | 5.7 | -16.8 | -16.7 |
| E141 | DL187009/CKSBL10153 | 3.2 | 5.8 | 5.2 | 4.7 | 2.2 | 7.9 | 7.6 | -9.5 | -5.9 |
| E142 | DL187009/CML71 | 3.4 | 5.8 | 5.5 | 4.9 | 2.3 | 11.1 | 5.6 | -13.3 | -9.0 |
| E143 | DL187009/CML370 | 3.3 | 6.0 | 5.6 | 5.0 | 1.9 | 5.3 | 5.2 | -11.6 | -3.2 |
| E144 | DL187010/DL187011 | 3.2 | 5.8 | 5.7 | 4.9 | 2.0 | 5.2 | 5.0 | -11.5 | -3.1 |
| E145 | DL187010/DL187012 | 3.5 | 6.1 | 5.9 | 5.2 | 2.4 | 6.3 | 3.4 | -14.4 | -4.5 |
| E146 | DL187010/DL187019 | 3.0 | 5.5 | 5.5 | 4.7 | 1.6 | 6.1 | 4.1 | -12.1 | -3.8 |
| E147 | DL187010/CML334 | 3.7 | 6.0 | 5.7 | 5.1 | 1.6 | 5.6 | 5.8 | -11.3 | -3.1 |
| E148 | DL187010/CKSBL10153 | 3.3 | 5.7 | 5.3 | 4.8 | 1.8 | 4.1 | 5.8 | -9.8 | -2.1 |
| E149 | DL187010/CML71 | 3.3 | 5.9 | 5.6 | 4.9 | 2.1 | 8.3 | 3.5 | -14.3 | -6.3 |
| E150 | DL187010/CML370 | 3.1 | 5.7 | 5.6 | 4.8 | 1.8 | 4.7 | 4.1 | -11.9 | -2.7 |
| E151 | DL187011/DL187012 | 3.7 | 6.4 | 5.8 | 5.3 | 1.7 | 9.7 | 3.8 | -15.5 | -6.9 |
| E152 | DL187011/DL187019 | 3.4 | 5.6 | 5.0 | 4.7 | 1.6 | 3.8 | 4.4 | -10.9 | -1.8 |
| E153 | DL187011/CML334 | 3.7 | 6.0 | 6.0 | 5.2 | 1.7 | 7.7 | 5.0 | -13.4 | -5.0 |
| E154 | DL187011/CKSBL10153 | 3.4 | 5.9 | 5.3 | 4.8 | 1.7 | 2.5 | 6.5 | -8.7 | -0.3 |
| E155 | DL187011/CML71 | 3.2 | 5.8 | 5.5 | 4.8 | 1.6 | 1.7 | 4.1 | -10.6 | 0.3 |
| E156 | DL187011/CML370 | 3.2 | 5.8 | 5.7 | 4.9 | 1.7 | 2.4 | 4.5 | -10.8 | -0.3 |
| E157 | DL187012/DL187019 | 3.2 | 5.8 | 5.7 | 4.9 | 1.9 | 6.2 | 4.1 | -12.7 | -4.1 |
| E158 | DL187012/CML334 | 3.6 | 5.9 | 5.3 | 4.9 | 2.0 | 11.7 | 4.0 | -15.1 | -9.4 |
| E159 | DL187012/CKSBL10153 | 3.6 | 5.9 | 5.4 | 5.0 | 1.9 | 7.0 | 7.7 | -9.7 | -4.6 |
| E160 | DL187012/CML71 | 3.4 | 6.0 | 5.5 | 5.0 | 1.9 | 4.7 | 3.7 | -12.7 | -2.7 |
| E161 | DL187012/CML370 | 3.2 | 5.7 | 5.8 | 4.9 | 1.7 | 2.9 | 5.7 | -9.9 | -0.7 |
| E162 | DL187019/CML334 | 3.3 | 5.9 | 5.4 | 4.9 | 2.1 | 9.0 | 5.3 | -12.6 | -6.9 |
| E163 | DL187019/CKSBL10153 | 3.1 | 5.4 | 5.3 | 4.6 | 1.7 | 2.5 | 5.1 | -9.3 | -0.5 |
| E164 | DL187019/CML71 | 2.9 | 5.7 | 5.5 | 4.7 | 1.7 | 3.7 | 4.3 | -11.0 | -1.7 |
| E165 | DL187019/CML370 | 2.9 | 5.8 | 5.5 | 4.7 | 1.9 | 9.2 | 4.7 | -12.8 | -6.9 |
| E166 | CML334/CKSBL10153 | 3.6 | 5.9 | 5.5 | 5.0 | 2.3 | 7.7 | 3.8 | -14.1 | -5.9 |
| E167 | CML334/CML71 | 3.7 | 5.8 | 5.3 | 4.9 | 1.8 | 6.1 | 5.8 | -11.0 | -3.9 |
| E168 | CML334/CML370 | 3.3 | 6.0 | 5.7 | 5.0 | 2.2 | 9.5 | 5.7 | -12.7 | -7.4 |
| E169 | CKSBL10153/CML71 | 3.2 | 5.7 | 5.3 | 4.7 | 2.1 | 6.6 | 5.2 | -11.4 | -4.7 |
| E170 | CKSBL10153/CML370 | 3.0 | 5.9 | 5.4 | 4.8 | 2.0 | 4.6 | 4.9 | -11.0 | -2.7 |
| E171 | CML71/CML370 | 3.7 | 6.2 | 5.6 | 5.2 | 1.9 | 4.3 | 4.3 | -12.6 | -2.2 |
| E172 | CML247/CML345 | 3.8 | 6.4 | 5.6 | 5.3 | 1.6 | 3.5 | 4.1 | -12.7 | -1.0 |
| E173 | CML269/CML345 | 3.4 | 5.8 | 5.7 | 5.0 | 1.8 | 6.4 | 4.9 | -12.2 | -4.0 |
| E174 | CML17/CML345 | 3.5 | 6.1 | 5.7 | 5.1 | 2.0 | 8.2 | 4.2 | -14.0 | -5.9 |
| E175 | CML23/CML345 | 3.3 | 5.9 | 5.6 | 4.9 | 2.3 | 10.4 | 6.0 | -12.6 | -8.3 |
| E176 | CML274/CML345 | 3.5 | 6.0 | 5.7 | 5.1 | 2.0 | 10.9 | 6.5 | -12.7 | -8.4 |
| E177 | CML371/CML345 | 3.5 | 6.1 | 5.9 | 5.1 | 2.3 | 14.0 | 5.2 | -15.5 | -11.6 |
| E178 | CML372/CML345 | 3.4 | 6.0 | 5.8 | 5.1 | 2.0 | 7.4 | 6.0 | -11.8 | -5.2 |
| E179 | CML402/CML345 | 3.2 | 6.0 | 5.3 | 4.8 | 1.9 | 6.6 | 5.2 | -11.6 | -4.5 |
| E180 | CML476/CML345 | 3.5 | 5.5 | 5.7 | 4.9 | 2.1 | 8.8 | 5.4 | -12.5 | -6.8 |
| E181 | DL187009/CML345 | 3.3 | 5.7 | 5.5 | 4.8 | 2.1 | 7.5 | 7.0 | -10.2 | -5.5 |
| E182 | DL187010/CML345 | 3.6 | 6.0 | 5.7 | 5.1 | 1.8 | 4.3 | 5.3 | -11.3 | -2.0 |
| E183 | DL187011/CML345 | 3.2 | 5.8 | 5.2 | 4.7 | 1.8 | 5.2 | 5.7 | -10.3 | -3.1 |
| E184 | DL187012/CML345 | 3.5 | 5.8 | 5.4 | 4.9 | 2.1 | 8.5 | 4.3 | -13.5 | -6.4 |
| E185 | DL187019/CML345 | 3.3 | 5.5 | 5.5 | 4.8 | 1.9 | 7.3 | 5.7 | -11.2 | -5.2 |
| E186 | CML334/CML345 | 3.7 | 6.0 | 5.3 | 5.0 | 1.9 | 9.8 | 5.7 | -12.9 | -7.4 |
| E187 | CKSBL10153/CML345 | 3.4 | 6.1 | 5.8 | 5.1 | 1.8 | 7.9 | 5.3 | -12.8 | -5.4 |
| E188 | CML71/CML345 | 3.7 | 6.0 | 5.2 | 5.0 | 2.5 | 20.5 | 3.5 | -19.3 | -18.1 |
| E189 | CML543/CKL05007/CML536 | 4.0 | 6.7 | 6.1 | 5.6 | 2.6 | 20.0 | 5.1 | -19.3 | -17.2 |
| E190 | CML444/CML442//CML543/CKL05017 | 3.6 | 6.7 | 6.4 | 5.5 | 2.4 | 18.4 | 5.9 | -17.6 | -15.4 |
| E191 | WH505 | 4.6 | 7.3 | 6.9 | 6.2 | 2.7 | 31.3 | 4.4 | -26.3 | -27.6 |
| E192 | WH401 | 4.8 | 7.1 | 6.4 | 6.1 | 2.3 | 17.4 | 3.1 | -21.7 | -14.4 |
| Mean |  | 3.5 | 6.0 | 5.7 | 5.1 | 2.0 | 8.1 | 5.1 | -13.1 | -6.0 |
| LSD0.05 |  | 0.3 | 0.3 | 0.4 | 0.3 | 0.4 | 5.7 | 1.6 |  |  |
|  |  |  |  |  |  |  |  |  |  |  |
|  |  |  |  |  |  |  |  |  |  |  |

LD1, LD2, LD3 = Leaf feeding damage score at 7, 14, 21 days after infestation, respectively; ED = Ear damage; ER = Ear rot; GY = Grain yield; PC1BI = Principal component-based index 1; PC2BI = Principal component-based index 2.
